# A Phylogenomic Approach to Clarifying the Relationship of *Mesodinium* within the Ciliophora: A Case Study in the Complexity of Mixed-Species Transcriptome Analyses

**DOI:** 10.1093/gbe/evz233

**Published:** 2019-10-30

**Authors:** Erica Lasek-Nesselquist, Matthew D Johnson

**Affiliations:** 1 New York State Department of Health (NYSDOH), Wadsworth Center, Albany, New York; 2 Biology, Woods Hole Oceanographic Institution, Woods Hole, Massachusetts

**Keywords:** *Mesodinium*, Litostomatea, ciliate phylogenomics, mixed-species transcriptomes, sequence contamination

## Abstract

Recent high-throughput sequencing endeavors have yielded multigene/protein phylogenies that confidently resolve several inter- and intra-class relationships within the phylum Ciliophora. We leverage the massive sequencing efforts from the Marine Microbial Eukaryote Transcriptome Sequencing Project, other SRA submissions, and available genome data with our own sequencing efforts to determine the phylogenetic position of *Mesodinium* and to generate the most taxonomically rich phylogenomic ciliate tree to date. Regardless of the data mining strategy, the multiprotein data set, or the molecular models of evolution employed, we consistently recovered the same well-supported relationships among ciliate classes, confirming many of the higher-level relationships previously identified. *Mesodinium* always formed a monophyletic group with members of the Litostomatea, with mixotrophic species of *Mesodinium*—*M. rubrum*, *M. major*, and *M. chamaeleon*—being more closely related to each other than to the heterotrophic member, *M. pulex*. The well-supported position of *Mesodinium* as sister to other litostomes contrasts with previous molecular analyses including those from phylogenomic studies that exploited the same transcriptomic databases. These topological discrepancies illustrate the need for caution when mining mixed-species transcriptomes and indicate that identifying ciliate sequences among prey contamination—particularly for *Mesodinium* species where expression from stolen prey nuclei appears to dominate—requires thorough and iterative vetting with phylogenies that incorporate sequences from a large outgroup of prey.

## Introduction

*Mesodinium* represents a phylogenetically problematic taxon subject to long-branch attraction (LBA) artifacts in SSU and LSU rDNA gene trees and inconsistent placement in phylogenomic analyses (Johnson et al. 2004; Strüder-Kypke et al. 2006; Vd’ačný et al. 2011; Chen, Ma, et al. 2015; Chen, Zhao, et al. 2015; Gao et al. 2016; Lynn and Kolisko 2017; Lynn et al. 2018). Due to their rapid evolutionary rates, *Mesodinium* spp. are often excluded during phylogenetic reconstruction (Strüder-Kypke et al. 2006; Vd’ačný et al. 2011; Gao and Katz 2014). Although representative SSU rDNA sequences for *Mesodiniumrubrum* and *Mesodiniumpulex* share three molecular synapomorphies common to all ciliates of the class Litostomatea, they also have additional insertions, deletions, and substitutions not shared by other ciliates (Johnson et al. 2004; Strüder-Kypke et al. 2006; Bass et al. 2009; Herfort et al. 2011). Morphological and ultrastructural classifications are equally problematic, with the family Mesodiniidae being placed within the order Haptorida of the class Litostomatea based on mouth positioning and inconspicuous oral ciliature (Corliss 1979), separated from other litostomes in the order Cyclotrichiida Jankowski, 1980 due to the absence of a dorsal brush and cytopharyngeal rods (nematodesmata) (Krainer and Foissner 1990), or relegated to an uncertain taxonomic status due to these unique characters and their somatic ciliature (Lynn 1991; Adl et al. 2019). Recent ultrastructural analyses revealed the only feature uniting Mesodiniidae with Litostomatea was the ciliary transition region (Garcia-Cuetos et al. 2012; Moestrup et al. 2012; Nam et al. 2012, 2015).

*Mesodinium* species are nearly ubiquitous in coastal and estuarine environments (Taylor et al. 1971; Lindholm 1985; Crawford 1989; Stoecker et al. 2009) and either display different degrees of mixotrophy—as is the case for *M. rubrum*, *M. major*, and *M. chamaeleon*, or are heterotrophic—as is the case for *M. pulex and M. pupula* (Johnson and Stoecker 2005; Herfort et al. 2011; Johnson 2011a; Garcia-Cuetos et al. 2012; Moestrup et al. 2012; Moeller and Johnson 2018). The *M. rubrum* and *M. major* species complex not only sequester plastids and mitochondria from their cryptophyte prey but also retain the cryptophyte nucleus or kleptokaryon, which they circumscribe for their own metabolic purposes (Gustafson et al. 2000; Johnson et al. 2007; Kim et al. 2017). Thus, mixotrophic *Mesodinium* harbor nuclear material from their own micronucleus, macronuclei, and mitochondria as well as the mitochondria, plastids, and nuclei of their prey, which has the potential to confound any molecular phylogenetic investigation.

Recent phylogenomic analyses have capitalized on the massive sequencing efforts of the Marine Microbial Eukaryote Transcriptome Sequencing Project (MMETSP) (Keeling et al. 2014) to clarify deeper evolutionary relationships within the phylum Ciliophora. Although these analyses have produced similar results supporting several higher-level relationships—such as the split between Postciliodesmophorata (classes Heterotrichea and Karyolictidea) and Intramacronucleata (all other ciliates), and the monophylies of CONthreeP (classes Colpodea, Oligohymenophorea, Nassophorea, Prostomatea, Plagiopylea, and Phyllopharyngea), and SAL (classes Spirotrichea, Armophorea, and Litostomatea), the phylogenetic placement of *Mesodinium* has remained uncertain or was not considered (Gao and Katz 2014; Gentekaki et al. 2014, 2017; Chen, Ma, et al. 2015; Chen, Zhao, et al. 2015; Feng et al. 2015; Lynn and Kolisko 2017; Sun et al. 2017; Lynn et al. 2018; Jiang et al. 2019).

Here, we seek to clarify the phylogenetic affinity of *Mesodinium* with additional RNA-Seq data for *M. rubrum*, *M. major*, and *M. chamaeleon*. Our analyses reveal that extracting legitimate ciliate sequences from mixotrophic cultures requires intense scrutiny of each transcriptome with at least two rounds of tree-building to differentiate prey and ciliate sequences. Additionally, we identified a substantial degree of contamination in most—if not all—MMETSP ciliate libraries, which could lead to the spurious placement of taxa if not filtered appropriately. Although the ribosomal RNA genes of *Mesodinium* spp. are well documented to have elevated evolutionary rates and are subject to LBA (Johnson et al. 2004; Strüder-Kypke et al. 2006; Herfort et al. 2011), we hypothesize that broader phylogenomic analyses placing *Mesodinium* outside the Litostomatea are likely due to the incorporation of contaminating sequences (e.g., Chen, Zhao, et al. 2015; Lynn and Kolisko 2017; Lynn et al. 2018). Our results firmly support the sister relationship of *Mesodinium* to other litostomes, lend support to relationships among ciliate classes, and weigh in on other controversial issues, such as the phylogenetic placement of *Protocruzia* and Prostomatea, with the most complete representation of the ciliate classes (9/11) thus far (Adl et al. 2019).

## Materials and Methods

### Culturing

Cultures of *M.rubrum* (CCMP 2563) and its prey, *Geminigera cryophila* (CCMP 2564), were isolated from McMurdo Sound, Antarctica (Gustafson et al. 2000). *Mesodinium chamaeleon* (NRMC1501) was isolated from the Narrow River, Rhode Island in 2015 (Moeller and Johnson 2018). The *M. rubrum* cultures were maintained in F/2-Si media (Guillard 1975) in 32 PSU seawater at 4 °C and at 5 and 65 μmol photons m^−2^ s^−1^ light and fed *G.cryophila* (CCMP 2564) at a ratio of ∼5:1 when transferred, once per week. To collect cell material for RNA extractions, *M. rubrum* cultures were gently filtered on to 1 μm polycarbonate filters and flash frozen in liquid nitrogen for later analysis. The *M. chamaeleon* culture was maintained in filtered 32 PSU seawater at 18 °C and 4 μmol photons m^−2^ s^−1^ light (Moeller and Johnson 2018). It was fed the cryptophyte *Storeatula major* (strain “g”) at a ratio of ∼10:1 when transferred, once per week. To collect material for RNA extractions, *M. chamaeleon* cells were grown in 0.5-l polystyrene tissue flasks and were first washed on Transwell Plate (Corning) filter inserts (8 μm), then collected onto 5-μm polycarbonate (47 mm) filters and flash frozen in liquid nitrogen. This filtering process drastically reduces the amount of free-living prey in *Mesodinium* cultures (Peltomaa and Johnson 2017; Moeller and Johnson 2018). Cell material for *M. major* was collected from red water off the coast of Chile (Johnson et al. 2016) by filtering water through a 0.2-μm Sterivex filter (EMD Milliopre, Billerica, MA) and freezing it in liquid nitrogen.

### Sequencing

Frozen cell pellets were extracted for RNA using a standard Trizol procedure (Sambrook et al. 1989). Poly-A enriched libraries were generated with the KAPA-stranded RNA-Seq kit and 150-bp paired-end sequencing was performed on an Illumina HiSeq 4000 at the University of Georgia Genomics Genome Facility.

### Transcriptome Assembly

*Mesodinium rubrum* and *M. chamaeleon* transcriptomes and the *M. major* metatranscriptome were assembled de novo with Trinity v.2.0.2 or v.2.2.0 (Haas et al. 2013) after removing adaptors and low-quality reads with BBDuk from BBMap v.35.82 (https://sourceforge.net/projects/bbmap/; last accessed November 4, 2019). Proteins were initially predicted with TransDecoder v.2.0.1 (https://github.com/TransDecoder/TransDecoder; last accessed November 4, 2019) using the standard genetic code. Transcriptomes from pure-cultures of *G.cryophila* prey were also sequenced and assembled and proteins were predicted using the same strategy as above to identify contamination due to free-living cryptophyte cells and the kleptokaryon. *Geminigeracryophila* cultures were exposed to several different treatments (such as high and low light conditions) to ensure that we captured a robust representation of the complete transcriptome.

### Other Ciliate and Outgroup Sequences

Ciliate and outgroup sequences used in the final analyses (table 1) and all prior steps (supplementary table 1, Supplementary Material online) were obtained from the MMETSP project or from genomes obtained from NCBI. All proteins from MMETSP libraries as well as the transcriptome assembly of *M.pulex* were downloaded from iMicrobe (https://www.imicrobe.us/; last accessed November 4, 2019) between 2016 and 2018. Raw reads from *Entodinium caudatum*, *Childonella uncinata*, *Balantidium ctenopharyngodoni*, *Colpoda aspera*, *Cryptocaryon irritans*, *Pseudomicrothorax dubius*, *Furgasonia blochmanni*, *Nassula variabilis*, and *Heterometopus* sp. were downloaded from the Sequence read archive (SRA) database (table 1) and assembled with MEGAHIT v.1.2.4 under default parameters (Li et al. 2015).

**Table 1:** Ciliate and Outgroup Species Included in Strategy 1 and 2 Phylogenies

Species	Phylum	Ciliate Class/Outgroup	G/T	Source	Accession Number
*Mesodinium chamaeleon*	Ciliate	Putative litostome	T	This study	SRR9987797–9987804
*Mesodinium major*	Ciliate	Putative litostome	T	This study	SRR9988875–9988876
*Mesodinium rubrum*	Ciliate	Putative litostome	T	This study	SRR10126743–10126758
*Mesodinium pulex*	Ciliate	Putative litostome	T	MMETSP	MMETSP0467
*Heterometopus* sp.	Ciliate	Armophorea	G	SRA	SRR6033281
*Aristerostoma* sp.	Ciliate	Colpodea	T	MMETSP	MMETSP0125
*Colpoda aspera*	Ciliate	Colpodea	T	SRA	SRR1768440
*Platyophrya macrostoma*	Ciliate	Colpodea	T	MMETSP	MMETSP0127
*Blepharisma japonicum*	Ciliate	Heterotrichea	T	MMETSP	MMETSP1395
*Climacostomum virens*	Ciliate	Heterotrichea	T	MMETSP	MMETSP1397
*Fabrea salina*	Ciliate	Heterotrichea	T	MMETSP	MMETSP1345
*Stentor coeruleus*	Ciliate	Heterotrichea	T	NCBI	GCA_001970955.1
*Balantidium ctenopharyngodoni*	Ciliate	Litostomatea	T	SRA	SRR5896119
*Entodinium caudatum*	Ciliate	Litostomatea	T	SRA	SRR8478280
*Litonotus pictus*	Ciliate	Litostomatea	T	MMETSP	MMETSP0209
*Furgasonia blochmanni*	Ciliate	Nassophorea	T	SRA	SRR6754448
*Nassula variabilis*	Ciliate	Nassophorea	T	SRA	SRR6754446
*Pseudomicrothorax dubius*	Ciliate	Nassophorea	T	SRA	SRR6754450
*Ichthyophthirius multifillis*	Ciliate	Oligohymenophorea	G	NCBI	GCA_000220395.1
*Paramecium tetraurelia*	Ciliate	Oligohymenophorea	G	NCBI	GCA_000165425.1
*Pseudocohnilembus persalinus*	Ciliate	Oligohymenophorea	G	NCBI	GCA_001447515.1
*Tetrahymena thermophila*	Ciliate	Oligohymenophorea	G	NCBI	GCA_000189635.1
*Chilodonella uncinata*	Ciliate	Phyllopharyngea	G	SRA	SRR6195042
*Cryptocaryon irritans*	Ciliate	Prostomatea	T	SRA	SRR5100657
*Euplotes crassus*	Ciliate	Spirotrichea	T	MMETSP	MMETSP1380
*Euplotes focardii*	Ciliate	Spirotrichea	T	MMETSP	MMETSP0205
*Euplotes focardii*	Ciliate	Spirotrichea	T	MMETSP	MMETSP0206
*Euplotes harpa*	Ciliate	Spirotrichea	T	MMETSP	MMETSP0213
*Favella ehrenbergii*	Ciliate	Spirotrichea	T	MMETSP	MMETSP0123
*Favella taraikaensis*	Ciliate	Spirotrichea	T	MMETSP	MMETSP0434
*Favella taraikaensis*	Ciliate	Spirotrichea	T	MMETSP	MMETSP0436
*Pseudokeronopsis* sp.	Ciliate	Spirotrichea	T	MMETSP	MMETSP1396
*Pseudokeronopsis* sp.	Ciliate	Spirotrichea	T	MMETSP	MMETSP0211
*Strombidinopsis acuminatum*	Ciliate	Spirotrichea	T	MMETSP	MMETSP0126
*Strombidinopsis* sp.	Ciliate	Spirotrichea	T	MMETSP	MMETSP0463
*Strombidium inclinatum*	Ciliate	Spirotrichea	T	MMETSP	MMETSP0208
*Strombidium rassoulzadegani*	Ciliate	Spirotrichea	T	MMETSP	MMETSP0449
*Stylonychia lemnae*	Ciliate	Spirotrichea	G	NCBI	GCA_000751175.1
*Protocruzia adherens*	Ciliate	Incertae sedis	T	MMETSP	MMETSP0216
*Cryptosporidium parvum*	Apicomplexa	Outgroup	G	NCBI	GCA_000165345.1
*Eimeria tenella*	Apicomplexa	Outgroup	G	NCBI	GCA_000499545.1
*Heterocapsa arctica*	Dinoflagellate	Outgroup	T	MMETSP	MMETSP1441
*Heterocapsa rotundata*	Dinoflagellate	Outgroup	T	MMETSP	MMETSP0503
*Heterocapsa triquestra*	Dinoflagellate	Outgroup	T	MMETSP	MMETSP0448
*Heterosigma akashiwo*	Stramenopile	Outgroup	T	MMETSP	MMETSP0414
*Heterosigma akashiwo*	Stramenopile	Outgroup	T	MMETSP	MMETSP0415
*Phaeodactylum tricornutum*	Stramenopile	Outgroup	G	NCBI	GCA_000150955.2
*Phytophthora infestans*	Stramenopile	Outgroup	G	NCBI	GCA_000142945.1
*Toxoplasma gondii*	Apicomplexa	Outgroup	G	NCBI	GCA_000006565.2

Note.—G/T, genome or transcriptome; accession number indicates MMETSP, GenBank assembly, or SRA accession number.

### Identification of *Mesodinium* Sequences and Homologs in Other Ciliate and Outgroup Databases

As shown previously, a considerable proportion of mixotrophic *Mesodinium* transcriptomes derived from the expression of prey sequences and the stolen prey nuclei that *Mesodinium* cells harbor (Lasek-Nesselquist et al. 2015). Traditional BLAST searches recover few reliably assigned *Mesodinium* sequences due to this heavy contamination and the lack of representation from closely related species in public databases. Thus, removing prey and kleptokaryon sequences from *Mesodinium* libraries while maximizing the number of putative ciliate sequences retained becomes a nontrivial endeavor. Two strategies were employed to extract *Mesodonium* sequences from mixed-species transcriptomes and to identify the homologs of these sequences in other ciliate and outgroup databases. Strategy 1 identified contaminating sequences in the *M. rubrum* assembly by comparing it to the transcriptome of pure-culture *G.cryophila* prey. The sequences that remained after filtering were considered to be ciliate and used to query additional databases (including other *Mesodinium* libraries). Strategy 2 searched for ciliate sequences in all *Mesodinium* assemblies, which were then used to query other databases. We anticipated that Strategy 1 would caste a wide net (yielding more potential *Mesodinium* sequences), whereas Strategy 2 would provide more reliably assigned sequences.

#### Strategy 1

Cd-hit-2d v.4.5.7 (Li and Godzik 2006; Fu et al. 2012) identified the union of proteins in *M.rubrum* and pure-culture *G.cryophila* transcriptome assemblies at 80% identity and returned only sequences unique to the *M. rubrum* data set. Although a sequence from the *M. rubrum* library might match at 80% identify over its length to a prey counterpart, it would be designated as unique to the *M. rubrum* library (i.e., classified as ciliate) if it was longer than the prey sequence. We relaxed this parameter by allowing proteins from the *M. rubrum* library to be characterized as *G. cryophila* even if they were 150 amino acids longer than those from the prey data set, which increases the number of potential contaminants captured. Open reading frames (ORFs) were recalled on select contigs with the subroutine getorf from EMBOSS v.6.3.1 (Rice et al. 2000) and translation table 6 to enable read-through of TAA and TAG stop codons, which translate for tyrosine in the *Mesodinium* genetic code (Swart et al. 2016). An in-house Python script translated ORFs into amino acid sequences. Of the 1,564 contigs with recalled ORFs, 694 showed ORFs of increased length after being translated with the *Mesodinium* alternative code and 383 showed an increased length of 100 amino acids or more (supplementary table 2, Supplementary Material online). There were 109 putative *M. rubrum* proteins that returned annotation from the KofamKOALA server of KEGG, which performs searches against HMM profiles of KO group alignments (Kanehisa 2002; Aramaki et al. 2019; Kanehisa et al. 2019). These proteins served as a seed to extract homologs from additional ciliate libraries as well as outgroup databases.

Homologs to *M.rubrum* seed sequences were identified in *M. chamaeleon*, *M. major*, and *M. pulex* transcriptome assemblies via TBlastN searches (Altschul et al. 1990, 1997; Camacho et al. 2009) using an *E*-value cutoff of 10^−30^. As ciliates contain multiple paralogs, the four-to-six contigs that returned the top best blast hits for each *M. rubrum* sequence were extracted. Coding sequences were called with the ciliate genetic code (genetic code 6) and were required to be a minimum of 150 nucleotides in length by the getorf subroutine of EMBOSS. All sequences were translated with the *Mesodinium* genetic code by an in-house Python script and the longest protein from each contig was blasted against its *M. rubrum* homolog to confirm its identity. The same process was employed to extract all potential homologs from ciliate assemblies generated from SRA submissions. Proteins were translated with the ciliate genetic code (genetic code 6) by getorf. BlastP searches of *M.rubrum* seed sequences against the proteins from MMETSP ciliates and outgroups, as well as the proteomes of ciliates and outgroups obtained from NCBI identified additional homologs, retaining only those hits with an *E*-value of 10^−30^ or better.

#### Strategy 2

*Mesodinium rubrum*, *M. chamaeleon*, and *M. major* assemblies were queried against a ciliate–cryptophyte database (see ciliates and cryptophytes listed in supplementary table 1, Supplementary Material online) or the RefSeq and SwissProt databases using a BlastX search to identify ciliate sequences. Only contigs returning hits to proteins from a ciliate genome assembly (such as *Tetrahymena thermophila*) with *E*-values ≤ 10^−04^ were considered. Coding sequences were identified and translated by TransDecoder v.5.5.0 with the *Mesodinium* genetic code option. The *M. pulex* transcriptome assembly from MMETSP was queried against these proteins via a BlastX search. Contigs with *E*-values ≤ 10^−30^ were retained for protein prediction with TransDecoder and the *Mesodinium* genetic code. All *Mesodinium* proteins were submitted to KofamKOALA on the KEGG server for annotation. Annotated proteins having a Kofam *E*-value of 10^−30^ or better were used to identify homologs in additional ciliate and outgroup databases. Ciliate transcriptome/genome assemblies generated from SRA submissions were queried against these *Mesodinium* proteins via BlastX searches. TransDecoder v.5.5.0 predicted proteins using the ciliate genetic code on contigs returning hits with an *E*-value ≤ 10^−30^. Proteins from MMETSP ciliate and outgroup databases were queried against the annotated *Mesodinium* proteins via BlastP searches and retained if they returned a hit with an *E*-value of 10^−30^ or better. Proteins were grouped together by their KO number, which yielded 610 KO numbers that contained at least one species of *Mesodinium*, one ciliate from the MMETSP database, and an outgroup. Of these, 378, which contained 24 or more sequences from the MMETSP and *Mesodinium* sequence sets, 1 member from the SRA set, and at least 4 outgroup sequences, were chosen for further analysis. We chose these requirements in order to retain a large number of alignments with adequate ciliate representation for meaningful phylogenetic placement of *Mesodinium*. At the same time, these requirements excluded alignments that contained several hundred to thousands of potential homologs, which would be difficult to visually inspect.

### Phylogenetic Reconstruction

#### Strategy 1

Ciliate and outgroup sequences were aligned in MAFFT v.7.058 (Katoh and Standley 2013) and visually inspected in MEGA v.70.26 (Kumar et al. 2016). Short sequences that did not overlap considerably with the rest of the alignment and obvious spurious sequences were removed as were the 5′ and 3′ ends of the alignment. Genealogies were reconstructed in FastTree v.2.1.8 (Price et al. 2010). Only ciliate sequences that formed monophyletic groups were extracted from each alignment (e.g., supplementary figs. 1 and 2, Supplementary Material online) along with appropriate outgroup sequences (those most closely related to the ciliate clade) from apicomplexans, dinoflagellates, and stramenopiles. Several genealogies contained multiple paralogs, which were separated into individual alignments if each set of paralogs formed a monophyletic group. Only one set of in-paralogs was considered if all in-paralogs recovered the same relationships (e.g., if two species contained multiple in-paralogs, where paralogs within each species formed a monophyletic group and each paralogous sequence set showed the same relationships among species, e.g., supplementary fig. 1, Supplementary Material online). Only the longest sequences were considered for taxa with multiple in-paralogs that clustered with each other (e.g., of in-paralogs, see supplementary figs. 1 and 2, Supplementary Material online). These alignments were further trimmed of ambiguous positions with trimAl v.1.2 (Capella-Gutiérrez et al. 2009) by considering only positions present in 60% or more of the sequences. Considerable contamination of MMETSP ciliate transcriptomes as well as all *Mesodinium* libraries became apparent after initial phylogenetic reconstruction with an outgroup containing broad taxonomic representation as well as further refinement with a second round of tree-building (e.g., supplementary figs. 1 and 2, Supplementary Material online). Although the goal of this article was to phylogenetically place *Mesodinium* with multiple alignments of high-confidence ciliate proteins, the iterative tree-building process removed *Mesodinium* representation from several of these alignments, where *Mesodinium* clearly grouped with prey clades or their phylogenetic placement was too uncertain to consider (e.g., supplementary fig. 2, Supplementary Material online). This left 42/73 alignments with at least one *Mesodinium* species represented (supplementary table 3, Supplementary Material online). The 73 protein alignments were concatenated into a supermatrix with an in-house Python script. The LG amino acid substitution model with a proportion of invariant sites and site-rates modeled by a relaxed gamma distribution (free rates; Yang 1995; Soubrier et al. 2012) with four rate categories (LG + I + R4) was applied in IQ-TREE v.1.6.9 (Nguyen et al. 2015) to generate a maximum likelihood phylogeny. To determine the possible effects of LBA on tree topology, site-rates were calculated in IQ-TREE and ML analyses were rerun with the fastest evolving sites successively removed from the alignment (site-rates ≥ 2, ≥1, and ≥0.8). Additionally, we ran our analyses under the LG + C20 + F amino acid mixture model (Si Quang et al. 2008), which represents an ML equivalent to the CAT model implemented in a Bayesian framework (Lartillot and Philippe 2004), to account for compositional heterogeneity. Support values for each tree were generated by the ultrafast bootstrap method with 1,000 replicates (Minh et al. 2013).

#### Strategy 2

MAFFT generated alignments for 378 KO sequence sets. FastTree reconstructed initial phylogenies to identify monophyletic ciliate groups and appropriate outgroup sequences from representatives of dinoflagellates, stramenopiles, and apicomplexans (table 1). When alignments contained multiple paralogs, only the monophyletic group with the greatest ciliate taxonomic representation was chosen and only if in-paralogs recovered the same phylogenetic relationships. We confirmed that the monophyletic group chosen consisted of ciliate sequences rather than a potential clade of contaminants by requiring the inclusion of least one sequence from a ciliate genome (table 1; e.g., supplementary figs. 1 and 2, Supplementary Material online). The longest sequence from each species was selected if multiple, highly similar sequences or in-paralogs were present. The final protein set consisted of 184 alignments that met these criteria (supplementary tables 4 and 5, Supplementary Material online). All monophyletic ciliate sequences and appropriate outgroup sequences were extracted from the initial alignments and realigned in MAFFT. The automated1 option in trimAl removed ambiguous positions after manually trimming 5′ and 3′ ends in MEGA. An in-house Python script created a supermatrix of all 184 protein alignments and a reduced supermatrix of 45 protein alignments. The alignments of the reduced supermatrix were required to contain >80% of ciliate taxa (30–36 out of 36 ciliates; supplementary table 5, Supplementary Material online) to examine the potential bias imposed by missing data. Maximum likelihood phylogenies were reconstructed for the complete and reduced supermatrices with the LG + I + R4 + F model in IQ-TREE. To mitigate the potential effects of LBA: 1) site-rates were calculated in IQ-TREE with the fastest evolving sites successively removed from the complete alignment (sites with rates ≥ 2 and sites with rates ≥1) and 2) the mixture model LG + C20 + F was employed in IQ-TREE to generate a ML phylogeny from the supermatrix with fast-evolving sites removed (site-rates ≥ 1). Branch support was assessed via the ultrafast bootstrap method with 1,000 replicates for all phylogenies.

### Topology Tests

Bootstrap proportion (BP) (Kishino et al. 1990), Shimodaira–Hasegawa (SH) (Shimodaira and Hasegawa 1999), Kishino–Hasegawa (KH) (Kishino and Hasegawa 1989), and approximately unbiased (AU) (Shimodaira 2002) topology tests were conducted in IQ-TREE v.1.6.9 using the RELL method of resampling (Kishino et al. 1990). We tested three hypotheses regarding the phylogenetic placement of *Mesodinium*: 1) *Mesodinium* as sister to Litosomatea in a SAL clade, 2) *Mesodinium* as sister to all other ciliates, and 3) *Mesodinium* as sister to Intramacronucleata. These relationships were recovered by our analyses and/or were recovered from previous analyses (e.g., Johnson et al. 2004; Strüder-Kypke et al. 2006; Gao et al. 2016; Lynn and Kolisko 2017; Lynn et al. 2018). Topology tests were conducted with Strategy 1 and Strategy 2 supermatrices. We also evaluated the position of *Protocruzia adherens* and the relationships of mixotrophic *Mesodinium* species, the only variably situated branches among all trees generated from Strategies 1 and 2. All trees were viewed in FigTree v.1.4.4 (http://tree.bio.ed.ac.uk/software/figtree/; last accessed November 4, 2019).

## Results

### Assembly Results

After quality filtering, the *M.rubrum*, *M. chamaeleon*, and *G.cryophila* libraries, each retained ∼80% of their paired-end reads, which generated >200,000–300,00 contigs (supplementary table 6, Supplementary Material online). Excluding contigs below 500 nucleotides, TransDecoder predicted 85,221 and 121,434 proteins from the *M. rubrum* and *G. cryophila* assemblies, with cryptophyte proteins representing at least 80% of the *M. rubrum* library. TransDecoder predicted 166,729 proteins from the *M. chamaeleon* transcriptome and of those with a BLAST hit against a ciliate–cryptophyte database, nearly 87% returned a hit to a cryptophyte. The *M.major* metatranscriptome retained 73% of its paired-end reads after quality filtering, which produced 284,200 contigs from a diverse eukaryotic community (supplementary table 6, Supplementary Material online). TransDecoder predicted 81,751 proteins—36,481 of which returned a BLAST hit against the RefSeq database. The vast majority (97%) returned best BLAST hits to nonalveolate species. MEGAHIT assembly results varied depending on the size and nature (RNA-Seq vs. DNA-Seq) of the library (supplementary table 6, Supplementary Material online).

### Phylogenies

The Strategy 1 and Strategy 2 data sets contained information for nine outgroup species (3 apicomplexans, 3 dinoflagellates, and 3 stramenopiles) and 35 and 36 ciliates, respectively, with representation across 9 ciliate classes: Heterotrichea, Spirotrichea, Armophorea, Litostomatea, Colpodea, Oligohymenophorea, Nassophorea, Phylopharyngea, and Prostomatea (table 1). The final Strategy 1 alignment consisted of 48,447 unambiguously aligned positions with 34,508 being parsimony-informative. The final Strategy 2 alignment consisted of 73,690 unambiguously aligned positions of which, 58,597 were parsimony-informative. Regardless of the strategy or model of evolution employed, the topology of the ciliate tree as well the generally strong node support remained remarkably consistent. The average proportion of missing data per taxon was 39% for the Strategy 1 supermatrix (supplementary table 7, Supplementary Material online). The average proportions of missing data for Strategy 2 complete and reduced-protein supermatrices were 39% and 25% per taxon, respectively (supplementary table 7, Supplementary Material online). In comparison, the average proportion of missing data for all *Mesodinium* species was 63% for Strategy 1 and 2 complete data sets and 57% for the reduced Strategy 2 alignment (supplementary table 7, Supplementary Material online).

#### Strategy 1

**Fig. 1.**
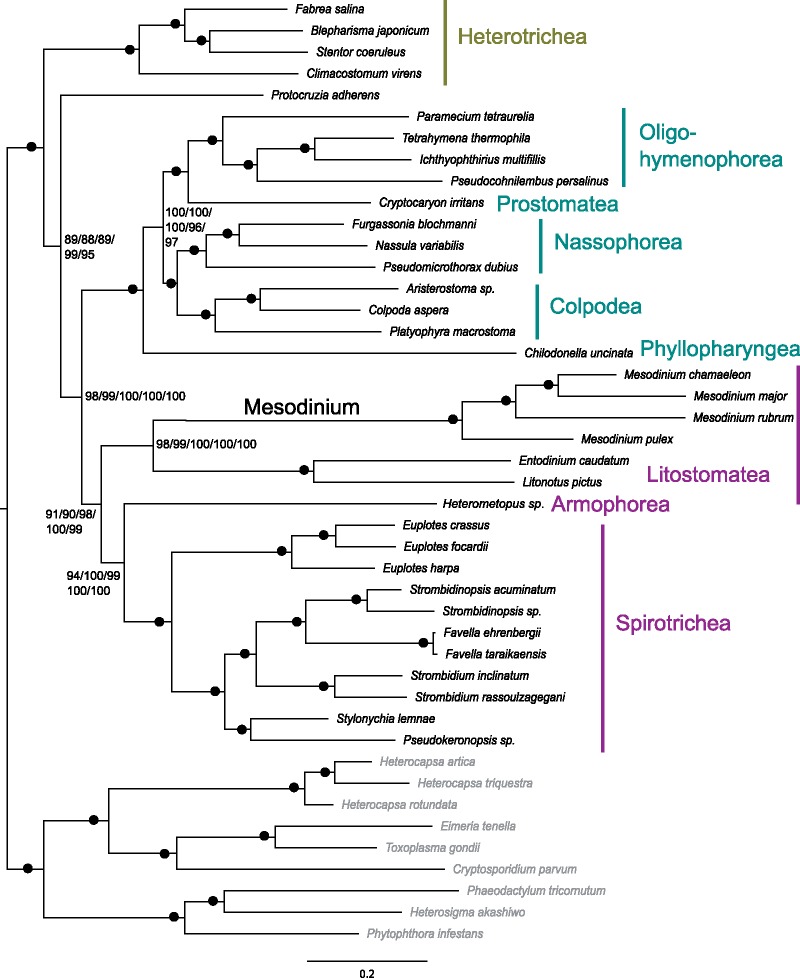
—Strategy 1 ML phylogeny of relationships among ciliates. Bootstrap support values are shown for the LG + I + R4 (1) and LG + C20 + F (2) models of evolution applied to the complete Strategy 1 supermatrix, and the LG + I + R4 model applied to Strategy 1 supermatrices with fast-evolving sites successively removed (3, 4, 5). Bootstrap values of 100% for all trees are indicated by a dot. Classes that belong to SAL, CONthreeP, and members of the heterotrichs (representing Postciliodesmophorata) are labeled in purple, blue, and green, respectively. Outgroup taxa are in gray.

All Strategy 1 trees recovered the same topology with strong bootstrap support (ranging from 89% to 100%) for all nodes (fig. 1). The heterotrichs (representing the Postciliodesmatophora) are sister to all other ciliates, whereas *P.adherens* (class Prostomatea) falls sister to the Intramacronucleata. All known classes with two or more representatives formed monophyletic groups, including the Heterotrichea, Oligohymenophorea, Nassophorea, Colpodea, Litostomatea, and Spirotrichea. The litostomes are sister to a clade comprised of Armophorea (represented by *Heterometopus*) + Spirotrichea, supporting the SAL consortium recovered by other phylogenomic data sets (Gentekaki et al. 2014, 2017; Lynn and Kolisko 2017; Lynn et al. 2018). The prostome, *C.irritans* falls at the base of the Oligohymenophorea, which is sister to a colpodid/nassophorid clade. *Chilodonella uncinata*, the one representative from the class Phyllopharyngea is sister to this larger clade, supporting the CONthreeP supergroup (Adl et al. 2019). *Mesodinium* forms a monophyletic group and clusters with the other two representatives of Listostomatea with strong bootstrap support (98–100%). Sequentially removing fast-evolving sites (leaving 26,753 final positions; supplementary table 8, Supplementary Material online) and applying an amino acid mixture model to mitigate the effects of LBA did not change the position of *Mesodinium*. Within the Mesodinidae, the same relationships were always recovered with 100% bootstrap support, where *M.rubrum* is sister to an *M. major* + *M. chamaeleon* clade and the heterotrophic *M. pulex* falls outside this mixotrophic group.

#### Strategy 2

**Fig. 2.**
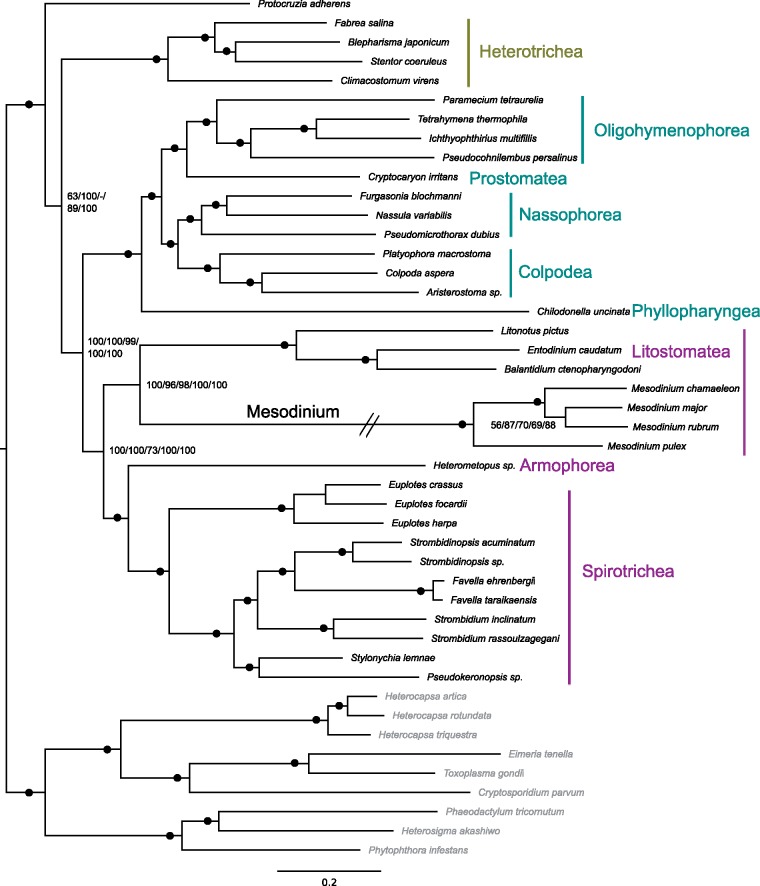
—Strategy 2 ML phylogeny of relationships among ciliates. Bootstrap support values are shown for the LG + I + R4 + F model of evolution applied to the complete Strategy 2 supermatrix (1), the LG + C20 + F model of evolution applied to the Strategy 2 supermatrix with fast-evolving sites removed (2), the LG + I + R4 + F model applied to a reduced-protein supermatrix (3), and the LG + I + R4 model applied to Strategy 2 supermatrices with fast-evolving sites successively removed (4, 5). Bootstrap values of 100% for all trees are indicated by a dot. Nodes not recovered by the reduced supermatrix are indicated with a dash. Classes that belong to SAL, CONthreeP, and members of the heterotrichs (representing Postciliodesmophorata) are labeled in purple, blue, and green, respectively. Outgroup taxa are in gray.

All Strategy 2 trees except for one recovered the same topology, which matched the relationships recovered by Strategy 1 except for the placement of *P.adherens* as sister to all other ciliates and the sister relationship between *M.major* and *M. rubrum* (fig. 2). The only tree that showed a deviation was generated by the reduced gene set, which showed *P. adherens* as sister to the Intramacronucleata—matching the topology of the Strategy 1 tree. Removing fast-evolving sites (leaving 43,818 final positions; supplementary table 9, Supplementary Material online) and employing a more sophisticated model of evolution did not alter the tree topology represented by the complete data set, rather it increased the support for existing nodes (fig. 2). Topology tests applied to the alignment with all fast-evolving sites removed indicated that trees where *P. adherens* was sister to all other ciliates and where *M. rubrum* was sister to *M. major* were significantly better than the alternatives (supplementary table 10, Supplementary Material online).

### Topology Tests for the Phylogenetic Placement of *Mesodinium*

Topology tests revealed that Strategy 2 alignments (complete and reduced) favored *Mesodinium* as sister to Litostomatea in a SAL supergroup to the significant exclusion of the other two topologies tested (table 2). Although Strategy 1 showed stronger support for this relationship as well, a topology where *Mesodinium* was sister to all other ciliates could not be excluded (table 2).

**Table 2:** Topology Tests for the Placement of Mesodiniidae within the Ciliate Tree

Topologies Tested	Log *L*	bp-RELL	KH	SH	AU
Strategy 1					
Litosomatea + *Mesodinium* sister to SA	−1,519,314.925	0.94	0.94	1	0.94
*Mesodinium* sister to all ciliates	−1,519,412.225	0.06	0.06	0.06	0.06
*Mesodinium* sister to Intramacronucleata	−1,519,518.003	0	0	0	7.48E−06
Strategy 2					
Litosomatea + *Mesodinium* sister to SA	−930,808.9543	1	1	1	1
*Mesodinium* sister to all ciliates	−931,224.529	0	0	0	1.24E−04
*Mesodinium* sister to Intramacronucleata	−931,059.5407	0	0	0	2.27E−06
Strategy2 reduced					
Litosomatea + *Mesodinium* sister to SA	−767,491.6799	0.96	0.96	1	0.96
*Mesodinium* sister to all ciliates	−767,576.2593	0.04	0.04	0.04	0.04
*Mesodinium* sister to Intramacronucleata	−767,612.8059	0	0.0013	0.0015	7.64E−04
32-Protein					
Litosomatea + *Mesodinium* sister to SA	−524,098.8587	0.07	0.07	0.08	0.07
*Mesodinium* sister to all ciliates	−524,038.8395	0.93	0.93	1	0.93
*Mesodinium* sister to Intramacronucleata	−524,176.735	0	0	0	6.21E−85

Note.—Three topologies were tested: 1) *Mesodinium* as sister to Litosomatea in the SAL supergroup as recovered for Strategy 1 and 2 trees, 2) *Mesodinium* as sister to Ciliophora as recovered by previous studies (e.g., Johnson et al. 2004) and the 32-protein supermatrix that included *Condylostoma magnum*, and 3) *Mesodinium* as sister to Intramacronucleata as recovered in Lynn and Kolisko (2017), Lynn et al. (2018), and others. Topologies were tested for Strategy 1 and 2 alignments (complete and reduced gene sets), and the 32-Protein supermatrix. Tests were performed in IQ-TREE using the RELL method of resampling with 10,000 resamplings. Log *L*, log likelihood; bp-RELL, bootstrap proportion using RELL method; KH, *P* value of one sided Kishino–Hasegawa test; SH, *P* value of Shimodaira–Hasegawa test; AU, *P* value of approximately unbiased test.

## Discussion

### Effects of Data Filtering and Refinement

Despite the large library sizes for *M.rubrum*, *M. chameleon*, and *M. major*, there were relatively few ciliate contigs assembled in comparison to cryptophyte prey, which dominated the transcriptomes. Even after *M. chamaeleon* cultures were washed using an 8.0 μm Transwell plate system (Moeller and Johnson 2018) and filtered using a relatively large pore size to remove the smaller prey cells, cryptophyte sequences still represented almost 90% of the predicted proteins in the ciliate assembly. These results are not surprising, considering that in all three species, prey nuclei are retained and transcriptionally active, along with other cryptophyte organelles (Gustafson et al. 2000; Johnson et al. 2007; Garcia-Cuetos et al. 2012; Moestrup et al. 2012; Kim et al. 2016, 2017). Thus, any analyses that do not include a mechanism to remove contamination are at risk of incorporating a large proportion of nonciliate sequences. This can lead to the spurious phylogenetic placement of *Mesodinium*, which might account for its sister relationship to the single prostome and heterotrich representatives in previous phylogenomic analyses (Chen, Zhao, et al. 2015; Lynn and Kolisko 2017; Lynn et al. 2018).

Both Strategy 1 and Strategy 2 relied on multipronged approaches to identify *Mesodinium* sequences among those of cryptophytes, with initial similarity searches (via Cd-Hit or BLAST), phylogenetic reconstruction with a large outgroup, and realignment of proteins confidently assigned as ciliate from the visual inspection of initial trees. Although Strategy 1 was employed to maximize the potential number of *Mesodinium* sequences identified in a time-efficient manner, it became apparent that whole-sale inclusion of ciliate sequences from this approach without phylogenetic assessment would yield too many false positives. Strategy 1 represented a subtraction method, where protein sequences from the *M.rubrum* library were removed if detected in the *G.cryophila* data set. However, proteins in the *M. rubrum* library 150 amino acids longer than similar sequences derived from the *G. cryophila* transcriptome would be classified as ciliate. This assumption led to the retention of contaminant sequences in the filtered *M. rubrum* data set and other *Mesodinium* data sets. Additionally, BLAST searches against the nr database revealed that some prey sequences were not present in the *G. cryophila* pure-culture transcriptome, leading to their classification as a ciliate. This suggests there are transcripts uniquely expressed by *G. cryophila* in a mixed-culture environment, possibly by the kleptokaryon, which would not be detected in comparison to a pure-culture library, regardless of its size and completeness. Thus, the filtering applied in Strategy 1 to remove cryptophyte contamination did not prevent the initial assignment of prey sequences as ciliate as indicated by the lack of *Mesodinium* species in 31 of 73 final alignments (where each alignment was required to contain at least one *Mesodinium* sequence initially). Strategy 2 identified fewer initial proteins as ciliate in the *Mesodinium* libraries but still yielded an adequate number of sequences for phylogenetic reconstruction and actually retained more proteins for final analyses than Strategy 1. The targeted approach of Strategy 2 was ultimately less labor intensive and is probably more appropriate for phylogenetic questions, where it is crucial to remove contamination.

The failure to account for the conservation among eukaryotic proteins or that eukaryotic prey and ciliates are often relatively closely related (e.g., alveolates and stramenopiles) hampers confident taxonomic assignment of sequences derived from mixed-species libraries. As demonstrated by our analyses, a simple BLAST approach to query any library derived from a mixed culture of ciliate and eukaryotic prey will recover multiple homologs from nonciliates without additional refinement, even when stringent cutoffs are applied (e.g., supplementary figs. 1 and 2, Supplementary Material online). We searched for prey 18s ribosomal rDNA sequences in ciliate MMETSP libraries to independently confirm the presence of contamination we had observed in our trees. In agreement with the results from our protein trees, we detected 18s rDNA sequences from prey as well as other microbial eukaryotes in the majority of the ciliate libraries tested, with the number of prey sequences sometimes outnumbering those from the ciliate (supplementary table 11, Supplementary Material online). Failure to account for this contamination most likely led to the association of *M. rubrum* with the prostome, *Tiarina fusus* (with 100% bootstrap support) and the paraphyletic nature of the *Mesodinium* clade in Chen, Zhao, et al. (2015). We found the *T.fusus* assembly to be heavily contaminated by prey and potentially other protists and we did not include this species in our final analyses (supplementary fig. 2 and table 11, Supplementary Material online). The results from our 18s rDNA analysis suggested that contamination from prey and other protists could represent up to 84% of the *T. fusus* library (supplementary table 11, Supplementary Material online; see supplementary table 12, Supplementary Material online, for an alignment between a full length contaminant contig from the *T. fusus* assembly and an 18s rDNA sequence from the Amoebozoa, *Vannella robusta*, which was not listed as the prey fed to *T. fusus*). There were 288 initial Strategy 2 alignments that contained *T. fusus* sequences but only 7 reliably recovered this taxon associated with a ciliate clade. A supermatrix for these proteins firmly grouped *T. fusus* with other members of CONthreeP (although not sister to *C.irritans*, the other prostome), whereas *Mesodinium* remained affiliated with Litostomatea (albeit with low-bootstrap support due to missing data; supplementary fig. 3, Supplementary Material online). Although the supermatrix is sparse for some taxa (particularly for *Mesodinium* species, which were only represented in one to three alignments) and only contained a few thousand characters, most of the higher-level relationships recovered by the complete data sets remained, suggesting that even a limited amount of highly curated data has the potential to provide phylogenetic insights.

The inclusion of a minor amount of contamination can alter topological outcomes if the contaminating genes contain enough phylogenetic information (Philippe et al. 2011; Brown and Thomson 2016). Although Lynn and Kolisko (2017) and Lynn et al. (2018) also employed a multistep process to remove paralogous and aberrant sequences from their phylogenomic data sets, they recovered *Mesodinium* outside the Intramacronucleata as sister to a heterotrich (albeit with low-bootstrap support). Lynn and Kolisko (2017) noted that for individual gene trees, *M. rubrum* fell outside a ciliate clade as frequently as it fell within one, a strong indication that its poorly supported position was due to conflicting signal from prey genes. This illustrates the need not only for a large eukaryotic outgroup that identifies broad-scale phylogenetic conflicts but for an outgroup that contains representative sequences from each prey species as well. Without cryptophyte representation in the initial gene trees used for filtering, the position of *M.rubrum* outside a ciliate clade could be interpreted as the effect of its fast-evolving nature rather than the presence of contamination. The likely erroneous positioning of *M. rubrum* is further perpetuated when different studies rely on the same data set to generate results (Gentekaki et al. 2014, 2017; Lynn and Kolisko 2017; Lynn et al. 2018).

Applying the appropriate genetic code was also critically important for improving the quality of data and the phylogenetic information available for *Mesodinium* species. A number of ciliates have been shown to have alternative genetic codes that reassign conserved eukaryotic stop codons to amino acids (Heaphy et al. 2016; Swart et al. 2016). Coding sequences translated with the standard genetic code instead of the *Mesodinium* code were on average 119–282 amino acids shorter (supplementary table 13, Supplementary Material online). However, the improvement in ciliate protein length due to the application of the proper code is probably greater than estimated because average differences were calculated for all coding sequences in the *Mesodinium* libraries, including those derived from cryptophytes. Indeed, when translated, select ORFs recalled with the *Mesodinium* genetic code were up to 4,000 amino acids longer than their counterparts called with the standard code (supplementary table 2, Supplementary Material online), reflecting the dramatic difference the appropriate code makes in data interpretation. Given that most ciliates appear to employ an alternative code, the quality of MMETSP data could also be improved by repredicting proteins (Swart et al. 2016). A simple approach to identifying proteins in EMBOSS (anything between a start and stop codon) with confirmation of protein identity by BLAST searches can also be an effective but cruder approach to extracting homologous proteins than more sophisticated programs. However, this approach might not be suitable for genomic data where the presence of introns could lead to truncated proteins. Overall, this approach identified fewer homologous sequences leading to the exclusion of *Balantidium ctenopharyngodoni* in Strategy 1.

### Phylogenies

Although Strategy 1 and Strategy 2 shared only nine proteins in common (supplementary table 4, Supplementary Material online), yielded supermatrices of considerably different lengths (48K vs. 73K characters), and contained multiple paralogs for the same gene (Strategy 1) or one representative (Strategy 2), they produced phylogenies with almost identical topologies.

Our results were also consistent with other phylogenomic analyses—robustly supporting a clade composed of Spirotrichea, Armophorea, and Litostomatea (SAL; Gentekaki et al. 2014, 2017; Lynn and Kolisko 2017; Lynn et al. 2018) and a clade composed of Colpodea, Oligohymenophorea, Nassophorea, and Phyllophyrangea (members of CONthreeP; Gao and Katz 2014; Lynn and Kolisko 2017; Lynn et al. 2018). Phyllopharyngea was sister to all other members of CONthreeP and Nassophorea + Colpodea were more closely related to each other than to Oligohymenophorea as previously observed in comparably large data sets (Lynn and Kolisko 2017; Lynn et al. 2018). Because our data sets provide the most complete taxonomic representations for CONthreeP (missing only the class Plagiopylea) and SAL (with seven litostomes in Strategy 2), we were able to provide some additional insights regarding higher-level relationships.

The addition of *C.irritans* to our data allowed us to interrogate the phylogenetic position of Prostomatea (Wright and Colorni 2002) within CONthreeP for the first time using a phylogenomic approach. Analysis of SSU and LSU rDNA genes is somewhat inconclusive regarding the phylogenetic relationships among Prostomatea, Oligohymenophorea, and Plagiopylea with studies recovering Plagiopylea + Prostomatea as sister to Oligohymenophorea (e.g., Lynn 2003; Strüder-Kypke et al. 2006; Miao et al. 2009; Vďačný et al. 2010; Zhang et al. 2012, 2014; Gao et al. 2016; Wang et al. 2017) or Plagiopylea as more closely related to Oligohymenophorea (e.g., Gong et al. 2009; Zhang et al. 2014; Chen, Ma, et al. 2015; Liu et al. 2015). The monophyly of Prostomatea has also been questioned (Zhang et al. 2012, 2014; Gao and Katz 2014; Liu et al. 2015). Although the phylogenomic analysis of Chen, Zhao, et al. (2015) included the prostome, *T.fusus*, only two other classes of CONthreeP (Colpodea and Oligohymenophorea) were represented. Moreover, the failure of *T. fusus* to group with these two classes was most likely the product of contamination and not necessarily indicative of its true affiliation, as discussed above. We recovered a sister relationship between *C. irritans* and Oligohymenophorea with full support regardless of the data set (Strategy 1 vs. Strategy 2, fast-evolving sites removed, or reduced gene data set) or model of evolution employed (LG models vs. a mixture model), confirming a close relationship between Prostomatea and Oligohymenophorea as proposed previously based on oral morphogenesis (Huttenlauch and Bardele 1987; Baroin-Tourancheau et al. 1992). Our supplementary analysis indicates that *T. fusus* is associated with CONthreeP but fails to recover Prostomatea as monophyletic supplementary fig. 3, Supplementary Material online). Further, the monophyletic clade composed of *Mesodinium* species and other litostomes rejects the idea that *Mesodinium* represents a separate class associated with Prostomatea and Plagiopylea (Zhang et al. 2012; Liu et al. 2015). Multigene data for Plagiopylea and additional data for Prostomatea would help to clarify the relationships of these two clades with each other and to the other members of CONthreeP.

Competing hypotheses for the relationships within SAL exist as well. Although analyses based on only a few molecules (predominantly SSU and LSU rDNA) frequently recover Armophorea and Litostomatea as sister taxa (Hammerschmidt et al. 1996; Strüder-Kypke et al. 2006; Gong et al. 2009; Miao et al. 2009; Vďačný et al. 2010; Zhang et al. 2012; Liu et al. 2015; Gao et al. 2016), phylogenomic analyses predominantly place Armophorea and Spirotrichea together (Gao and Katz 2014; Gentekaki et al. 2014, 2017; Lynn et al. 2018). However, with the exception of Gao and Katz (2014), these studies relied on the same core data set from Gentekaki et al. (2014). As our data sets share only 15/73 (Strategy 1 not including paralogs; supplementary table 3, Supplementary Material online) and 19/184 (Strategy 2; supplementary table 4, Supplementary Material online) proteins in common with Gentekaki et al. (2014), we provide independent support for the closer relationship between Armophorea and Spirotrichea and are in agreement with the conclusion that *Protocruzia* is not a member of Spirotrichea (Li et al. 2010; Gentekaki et al. 2014) but of uncertain standing within the Intramacronucleata (Adl et al. 2019). The position of *P.adherens* changes upon reducing the Strategy 2 data set to minimize missing data. Although simulation studies have highlighted biases introduced by missing data (Lemmon et al. 2009), some recent empirical analyses have demonstrated increased or unchanged phylogenetic accuracy when loci with missing data are retained (Wiens and Morrill 2011; Roure et al. 2013; Jiang et al. 2014; Streicher et al. 2016). Given the stability of the tree topologies otherwise, the phylogenetic placement of *P.adherens* seems influenced more by the dramatic reduction of phylogenetic information than the decrease in missing data. Although topology tests rejected the position of *P. adherens* recovered in Strategy 1 for a position at the root of the ciliate tree, the discrepant results generated by these data sets and others (e.g., supplementary fig. 3, Supplementary Material online) reflect the continued uncertainty surrounding the placement of *Protocruzia* and underscore the need for increased taxonomic sampling within this group.

Our results confidently and consistently recover a sister relationship between *Mesodinium* and Litosomatea (figs. 1 and 2) in contrast to SSU and LSU rDNA phylogenies (Johnson et al. 2004; Zhang et al. 2012; Chen, Ma, et al. 2015; Gao et al. 2016) or previous phylogenomic studies (Chen, Zhao, et al. 2015; Lynn and Kolisko 2017; Lynn et al. 2018). The presence of contamination has a major influence on phylogenetic outcomes (Philippe et al. 2011) but differences in taxonomic sampling could also contribute to the variable position of *Mesodinium*, particularly when comparing our results to those of Lynn and Kolisko (2017) and Lynn et al. (2018), which show *M.rubrum* as sister to Intramacronucleata with a heterotrich. We mimicked the taxonomic sampling of Lynn and Kolisko (2017) and Lynn et al. (2018) by removing all but one representative of the heterotrichs from our Strategy 2 data set and by including only *M. rubrum* and *Litonotus pictus* from the Litostomatea to determine whether we could recover the same relationship between *Mesodinium* and Heterotrichea. Despite the reduced taxonomic sampling, we recovered *M. rubrum* and *Litonotuspictus* as sister taxa in the same relationship to Armophorea and Spirotrichea as we had observed from our complete-taxon data set (supplementary fig. 4, Supplementary Material online). To determine the potential effect of specific taxa on the position of *Mesodinium*, we generated a supermatrix from all 32 Strategy 2 protein alignments that contained *Condylostoma magnum*—the heterotrich used in Lynn and Kolisko (2017) and Lynn et al. (2018). With only *Condylostomamagnum* to represent the heterotrichs, a single *Mesodinium* species fell at the base of the ciliate tree (supplementary fig. 5, Supplementary Material online). The position of *Mesodinium* remained unchanged despite successively adding back heterotrich and *Mesodinium* taxa to the data set (supplementary fig. 5, Supplementary Material online) or switching the single heterotrich representative (results not shown), which demonstrates that the number of proteins included in the analysis was probably more influential than specific taxa on topology. Although increased taxonomic sampling has been shown to increase clade support by breaking-up long branches (Graybeal 1998; Hedtke et al. 2006; Heath et al. 2008), this factor appears less relevant in positioning *Mesodinium* than the number of informative characters included in the analysis. The 32-protein supermatrix (containing 12,583 amino acid residues) failed to recover *Mesodinium* + Litostomatea even when the maximum number of heterotrichs, *Mesodinium* species, and litostomes were included. The complete character matrix of Strategy 2 (containing > 70,000 amino acid residues) recovered this relationship with full support despite minimizing the number of heterotrichs, *Mesodinium* species, and litostomes. Further, topology tests performed with the 32-protein supermatrix could not reject a phylogeny with *Mesodinium* as sister to Litostomatea as significantly worse than a phylogeny with *Mesodinium* as sister to all ciliates (table 2). Strategy 2 supermatrices rejected all topologies tested except the one with a sister relationship between *Mesodinium* and Litostomatea (table 2). This suggests the position of *Mesodinium* recovered by the 32-protein supermatrix is due to some conflicting signal that becomes swamped by the addition of more characters favoring the *Mesodinium* + Litostomatea relationship. The reduced supermatrix for Strategy 2 and the 32-protein supermatrix share 18 alignments in common (supplementary table 5, Supplementary Material online). The 14 alignments unique to the 32-protein supermatrix contained fewer ciliates (supplementary table 5, Supplementary Material online) and four had no *Mesodinium* representation, which contributed to >70% missing data for three of the four *Mesodinium* species in this protein set. Visual inspection revealed sequences of poorer quality (e.g., large insertions and truncated sequences leading to gappy alignments) with more ambiguously aligned positions than those of the 18 shared with Strategy 2. The 18 common alignments recovered a ML phylogeny identical to the reduced Strategy 2 tree, whereas the 14 alignments unique to the 32-protein supermatrix recovered *Mesodinium* at the base of the ciliate tree. Thus, it appears the 32-protein supermatrix tree is influenced by alignments of poorer quality, which are buffered by additional data in the Strategy 2 supermatrices.

Because more data can converge on a well-supported but incorrect topology due to model misspecification (Phillips et al. 2004; Delsuc et al. 2005; Hedtke et al. 2006; Philippe et al. 2011), we attempted to mitigate the effects of LBA by removing fast-evolving sites and applying a CAT-like mixture model to account for compositional biases in amino acid frequencies. The position of *Mesodinium* remained unaffected, indicating that the data were robust even with the inclusion of saturated sites and possible model misspecification, although removal of fast-evolving sites improved support for existing clades—particularly for the sister relationship between *M.major* and *M. rubrum* in Strategy 2 trees. The sister relationship between *M. rubrum* and *M. major* in the Strategy 2 trees is in agreement with other molecular studies (Garcia-Cuetos et al. 2012; Johnson et al. 2016) but in discord with Strategy 1, reflecting the close evolutionary relationships among *M. chamaeleon*, *M. rubrum*, and *M. major*. Although acquired phototrophy has arisen independently numerous times across the eukaryotic tree (Stoecker et al. 2009; Johnson 2011b; Selosse et al. 2017), the well-supported clade of *M. chamaeleon*, *M. rubrum*, and *M. major* suggests that the mixotrophic lifestyle arose once in *Mesodinium*.

## Conclusions

Phylogenomic analyses are lending confidence to deeper evolutionary relationships within Ciliophora as described initially by morphological, ultrastructural, and single-to-few gene studies. With two new phylogenomic data sets, we provide additional support for the CONthreeP and SAL supergroups—with increased taxonomic representation for both—and confirm the problematic nature of *Protocruzia*. That independent multiprotein analyses converge on many of the same results suggests these large-data approaches will help resolve other conflicts within the ciliate tree. Here, we find robust support for the monophyly of Mesodiniidae and its traditional affiliation with Litostomatea (Corliss 1979; Small and Lynn 1981), illustrating the promise phylogenomic analyses hold for elucidating the evolutionary relationships of problematic taxa and underscoring the crucial need for filtering contamination when working with ciliate or any mixed-culture transcriptomes.

## Supplementary Material

Supplementary data are available at *Genome Biology and Evolution* online.

## Supplementary Material

evz233_Supplementary_Data

## Notes

**Data deposition:** This project has been deposited in the NCBI SRA database under accessions PRJNA560206 (*Mesodinium rubrum and Geminigera cryophila*), PRJNA560220 (*Mesodinium chamaeleon*), and PRJNA560227 (*Mesodinium major*). All phylogenies and alignments included in this study have been deposited in Dryad: 10.5061/dryad.zw3r22848.
