# Supplementary material for: A Phylogenomic Approach to Clarifying the Relationship of Mesodinium within the Ciliophora: A Case Study in the Complexity of Mixed-Species Transcriptome Analyses
Source: Genome Biol Evol. 2019 Oct 30;11(11):3218–32. doi: 10.1093/gbe/evz233 (PMC6859813; doi:10.1093/gbe/evz233)
Supplement: evz233_Supplementary_Data [file evz233_supplementary_data.zip › Supporting_Information_Legends.pdf]

Supplementary Fig. 1. Initial phylogenetic tree for protein alignment K00600 with a large outgroup to identify contamination in ciliate libraries. Colored clades represent those of prey, which contain contaminating sequences from ciliate libraries. The contaminating sequences are labeled in purple within the prey clades. The clades highlighted in red, orange, green, blue, pink, and brown represent those of cryptophytes, dinoflagellates, chlorophytes, stramenopiles, haptophytes, and euglenozoa, respectively. Ciliate sequences in grey represent those of uncertain phylogenetic origin, being close sisters to or paraphyletic with a prey clade. The node highlighted with a purple "*" at the top of the tree represents ciliate sequences distantly related to other ciliate homologs, such as those found in the monophyletic ciliate group boxed in light purple. In-paralogs can be observed for several ciliate species within the monophyletic clade (ex. *Euplotes focardii*, *Heterometopus sp.*, *Pseudokeronopsis sp*., and *Favella taraikaensis*). There are two paralogous clades comprised of sequences from *Stylonychia lemnae*, *Pseudokeronpsis sp.*, *Strombidium rassoulzagegani*, *S. inclinatum*, *Strombidinopsis acuminatum*, and *Favella* spp. but the relationships among these species are the same for both clades (branches in white within the purple box). Species abbreviations (before the first underscore) are according to Table 1. Major eukaryotic groups are distinguished with a letter between two underscores as follows: a, apicomplexans; c, ciliates; d, dinoflagellates; e, euglenozoa; f, fungi; h, haptophytes; r, cryptophytes; s, stramenopiles; v, chlorophytes. Protein accession numbers proceed species and group identifications.

Supplementary Fig. 2. Initial phylogenetic tree for the protein alignment K01870, which shows the extent of contamination in the *Tiarina fusus* library. All K01870 sequences from the *T. fusus* library – labeled in purple – are contaminants. Contaminants group with cryptophytes (the prey of *T. fusus*) and stramenopiles. Cryptophyte and stramenopile clades that include contaminant

sequences are highlighted in red and blue, respectively. There are *T. fusus* sequences (labeled in grey) that form a close sister group to euglenozoa (highlighted in brown), representing another possible source of contamination. The tree also shows a monophyletic ciliate group – boxed in light purple - with multiple in-paralogs for several species (ex. *Paramecium tetraurelia*, *Aristerostoma sp*. and *Euplotes harpa*). The *Mesodinium major* sequence boxed in grey-purple falls sister to the ciliate monophyly as well as a large clade of outgroup sequences, making it an example of a *Mesodinium* sequence that cannot be confidently placed within the ciliate group. Species abbreviations (before the first underscore) are according to Table 1. Major eukaryotic groups are distinguished with a letter between two underscores as follows: a, apicomplexans; c, ciliates; d, dinoflagellates; e, euglenozoa; f, fungi; h, haptophytes; r, cryptophytes; s, stramenopiles; v, chlorophytes. Protein accession numbers proceed species and group identifications.

Supplementary Fig. 3. ML phylogeny showing the phylogenetic placement of *Tiarina fusus* (class Prostomatea). ML phylogeny generated with a 7 protein supermatrix under the LG+I+R4 model of evolution with 1000 ultrafast bootstrap replicates as implemented in IQ-TREE. The supermatrix includes all proteins for which *T. fusus* grouped with a ciliate monophyly. Members of CONthreeP (including the other prostome, *Cryptocaryon irritans*) are boxed in purple.

Supplementary Fig. 4. ML phylogeny with reduced taxonomic sampling for Heterotrichea, Litosomatea, and *Mesodinium*. ML phylogeny generated under an LG+I+R4 model of evolution with 1000 ultrafast bootstrap replicates as implemented in IQ-TREE. The supermatrix derives from Strategy 2 with all members of Heterotrichea, Litostomatea, and *Mesodinium* removed except for *S. coeruleus*, *L. pictus*, and *M. rubrum*, respectively. Despite recapitulating the

taxonomic sampling of Lynn and Kolisko (2017) and Lynn et al. (2018), we still recovered *Mesodinium* as sister to Litostomatea with full support.

Supplementary Fig. 5. ML phylogenies to demonstrate potential effects of taxon choice on topology. All phylogenies were generated from a 32 protein supermatrix that contained *C. magnum* as a representative heterotrich with the LG+I+R4 model of evolution and 1000 bootstrap replicates as implemented in IQ-TREE. a) ML phylogeny with only *C. magnum* and *M. major* as representative heterotrich and *Mesodinium* taxa, respectively, b) ML phylogeny with two heterotrich taxa (*C. magnum* and *C. virens*), c) ML phylogeny with three heterotrich taxa (*C. magnum*, *C. virens*, and *F. salina*), d) ML phylogeny with four heterotrich taxa (*C. magnum*, *C. virens*, *F. salina*, and *B. japonicum*), e) ML phylogeny with all heterotrich taxa, f) ML phylogeny with all heterotrich and *Mesodinium* taxa. Outgroup not shown for ease of visualization.
