# Supplementary figures and images for: A Phylogenomic Approach to Clarifying the Relationship of Mesodinium within the Ciliophora: A Case Study in the Complexity of Mixed-Species Transcriptome Analyses
Source: Genome Biol Evol. 2019 Oct 30;11(11):3218–32. doi: 10.1093/gbe/evz233 (PMC6859813; doi:10.1093/gbe/evz233)

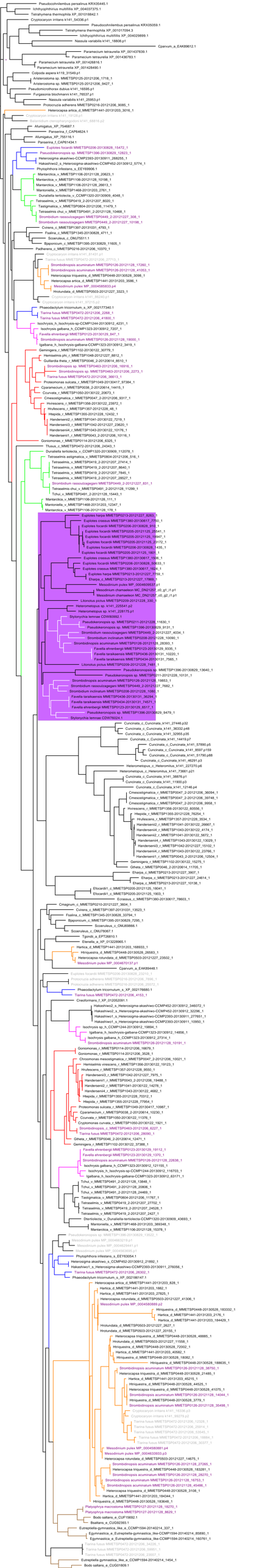

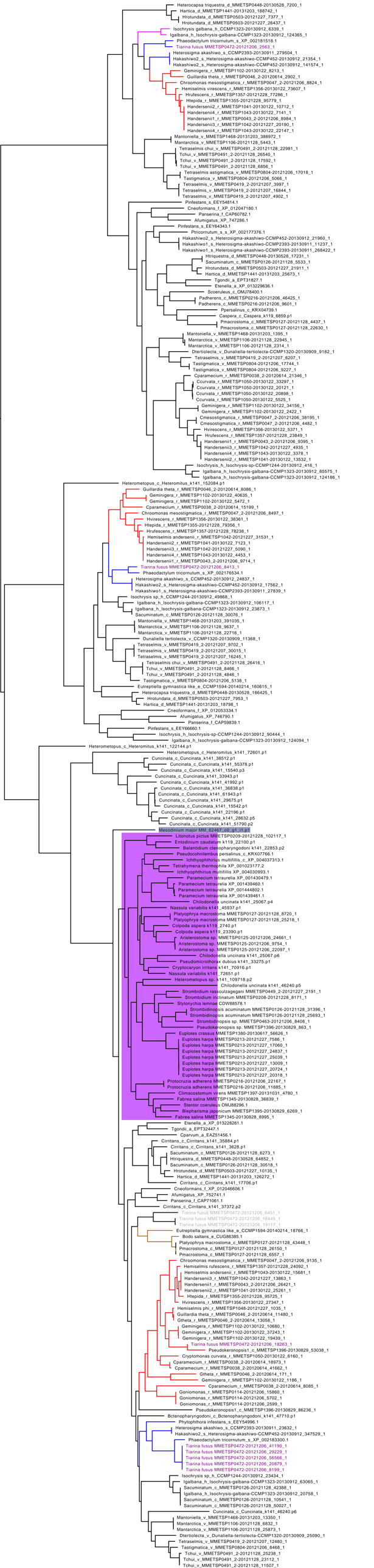

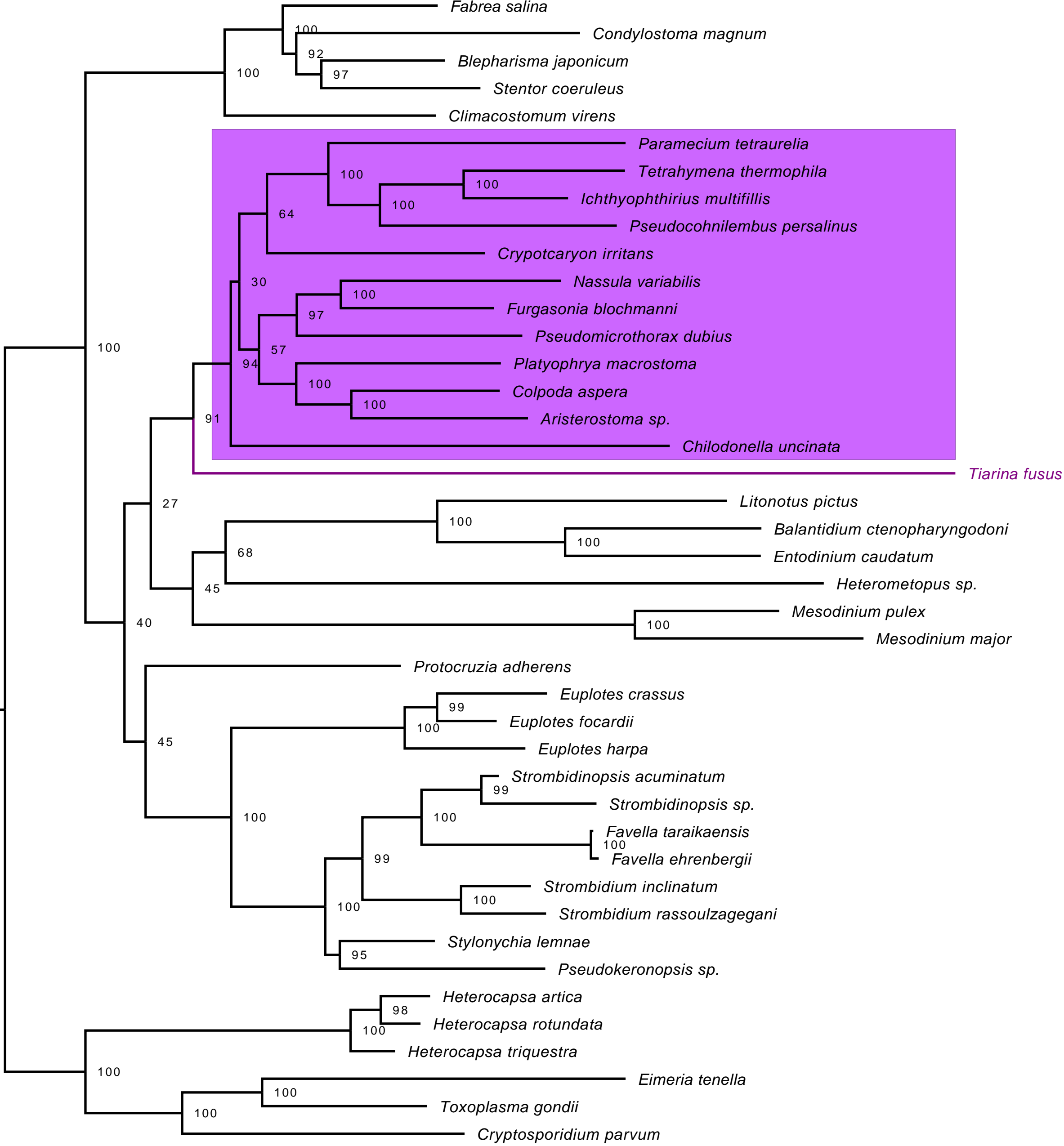

0.1

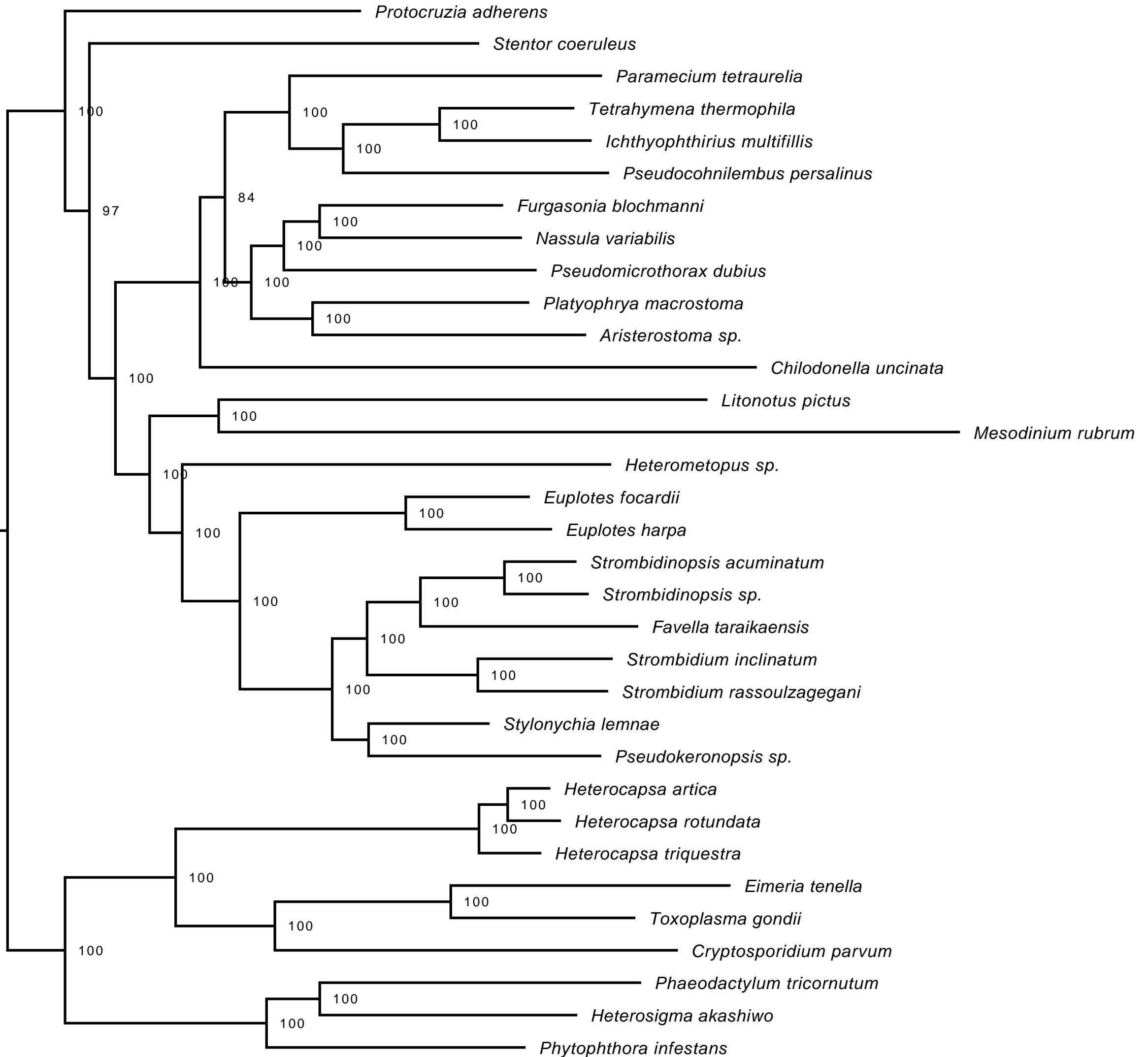

0.2

Supplementary Figure 5

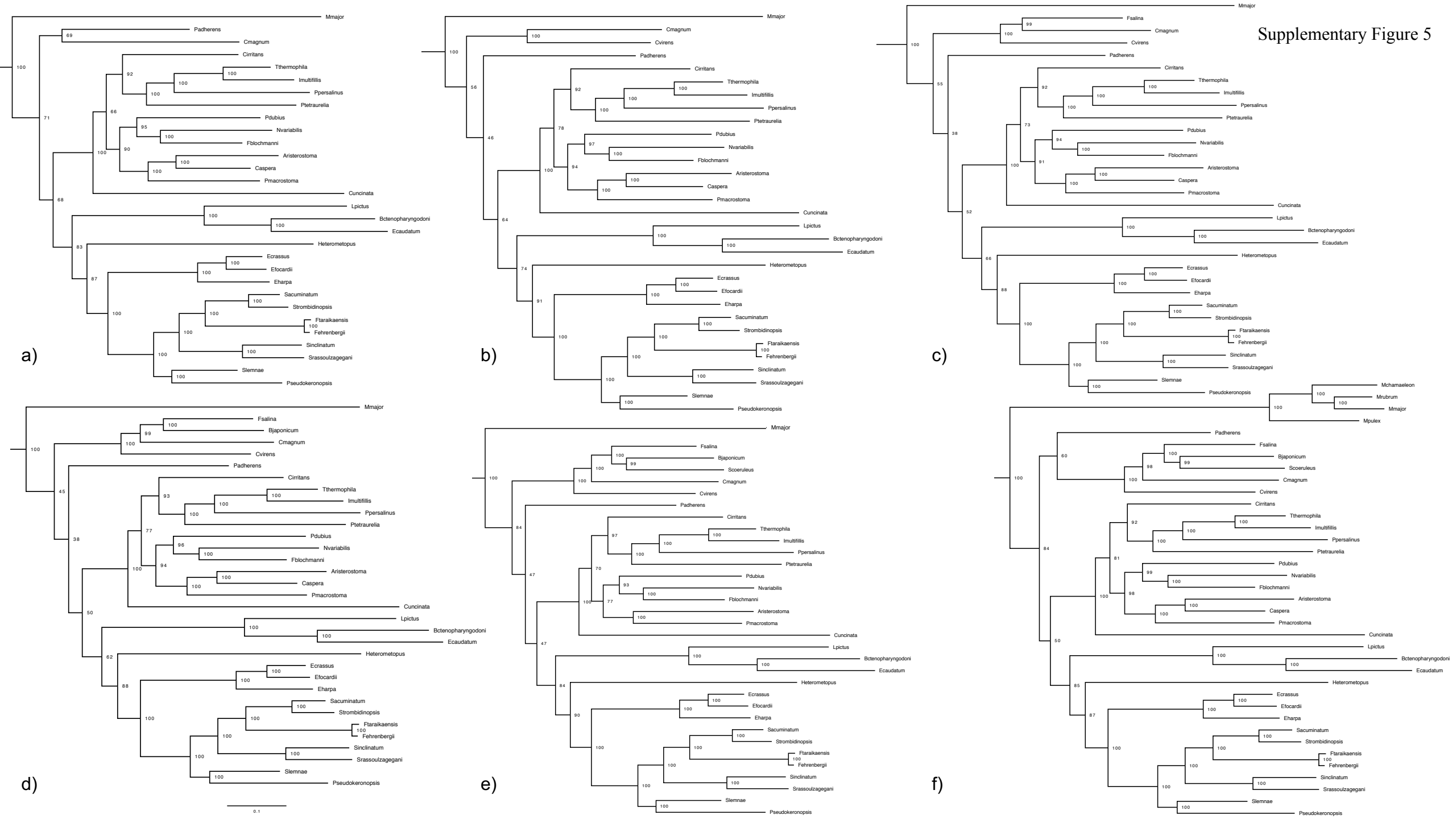

Supplement: evz233_Supplementary_Data [file evz233_supplementary_data.zip › SupplementaryFigures1-5.pdf]
